# Plant-based production of an orally active cyclotide for the treatment of multiple sclerosis

**DOI:** 10.1007/s11248-023-00341-1

**Published:** 2023-03-17

**Authors:** Mark A. Jackson, Jing Xie, Linh T. T. Nguyen, Xiaohan Wang, Kuok Yap, Peta J. Harvey, Edward K. Gilding, David J. Craik

**Affiliations:** Institute for Molecular Bioscience, Australian Research Council Centre of Excellence for Innovations in Peptide and Protein Science, The University of Queensland, Brisbane, QLD 4072 Australia

**Keywords:** Multiple sclerosis, *Nicotiana benthamiana*, Asparaginyl endopeptidases, Peptide, Recombinant, Plant molecular farming, Cyclotide

## Abstract

Multiple sclerosis (MS) is a debilitating disease that requires prolonged treatment with often severe side effects. One experimental MS therapeutic currently under development is a single amino acid mutant of a plant peptide termed kalata B1, of the cyclotide family. Like all cyclotides, the therapeutic candidate [T20K]kB1 is highly stable as it contains a cyclic backbone that is cross-linked by three disulfide bonds in a knot-like structure. This stability is much sought after for peptide drugs, which despite exquisite selectivity for their targets, are prone to rapid degradation in human serum. In preliminary investigations, it was found that [T20K]kB1 retains oral activity in experimental autoimmune encephalomyelitis, a model of MS in mice, thus opening up opportunities for oral dosing of the peptide. Although [T20K]kB1 can be synthetically produced, a recombinant production system provides advantages, specifically for reduced scale-up costs and reductions in chemical waste. In this study, we demonstrate the capacity of the Australian native *Nicotiana benthamiana* plant to produce a structurally identical [T20K]kB1 to that of the synthetic peptide. By optimizing the co-expressed cyclizing enzyme, precursor peptide arrangements, and transgene regulatory regions, we demonstrate a [T20K]kB1 yield in crude peptide extracts of ~ 0.3 mg/g dry mass) in whole plants and close to 1.0 mg/g dry mass in isolated infiltrated leaves. With large-scale plant production facilities coming on-line across the world, the sustainable and cost-effective production of cyclotide-based therapeutics is now within reach.

**Supplementary Information:**

The online version contains supplementary material available at 10.1007/s11248-023-00341-1.

## Introduction

Multiple sclerosis (MS) is a devastating autoimmune disease that affects the central nervous system leading to progressive neurological disability (Macaron and Ontaneda 2019). Disease onset typically manifests itself in young adults aged 20 – 30 years, where patients present with a relapsing-remitting disease often followed by a secondary progressive disease stage (McGinley et al. 2021). Although the exact cause of MS is unclear, genetic, and environmental factors have been implicated (Perez-Perez et al. 2022; Manouchehrinia et al. 2022; Leffler et al. 2022). Treatment options for MS have progressed in recent years, with broad-spectrum immunosuppressant approaches making way for more targeted immunotherapies that include recombinant interferons, e.g. IFNβ (Walther et al. 1996), small molecules, e.g. fingolimod (Pournajaf et al. 2022) and antibodies, e.g. natalizumab, alemtuzumab (Morrow et al. 2022). However, these therapeutics have varying efficiencies and deleterious side effects (Jarmolowicz et al. 2017; Moiola et al. 2020); thus, there is a clear need to develop novel, less toxic and easy-to-administer MS therapeutics.

Cyclotides are a class of backbone cyclic and disulfide bond stabilized plant-produced peptides that have attracted interest for applications in drug development (Camarero and Campbell 2019; Craik et al. 2012). The potential of cyclotides as a treatment for MS was first put forward by Grundemann et al. (2012), who demonstrated that a cyclotide enriched *Oldenlania affinis* plant extract had an antiproliferative effect on activated human lymphocytes. Subsequent fractionation activity assessments revealed that in isolation, the isolated cyclotide kalata B1 was antiproliferative in a dose-dependent manner. Furthermore, single residue substitutions were identified that conferred either pronounced or reduced T-cell proliferation suppression (Grundemann et al. 2013). Subsequent mode-of-action studies utilizing the [T20K]kalata B1 mutant revealed that this peptide influences the expression level of the interleukin 2-receptor (IL-2), a key component of T-cell receptor signaling (Grundemann et al. 2013; Hellinger et al. 2021).

**Fig. 1:**
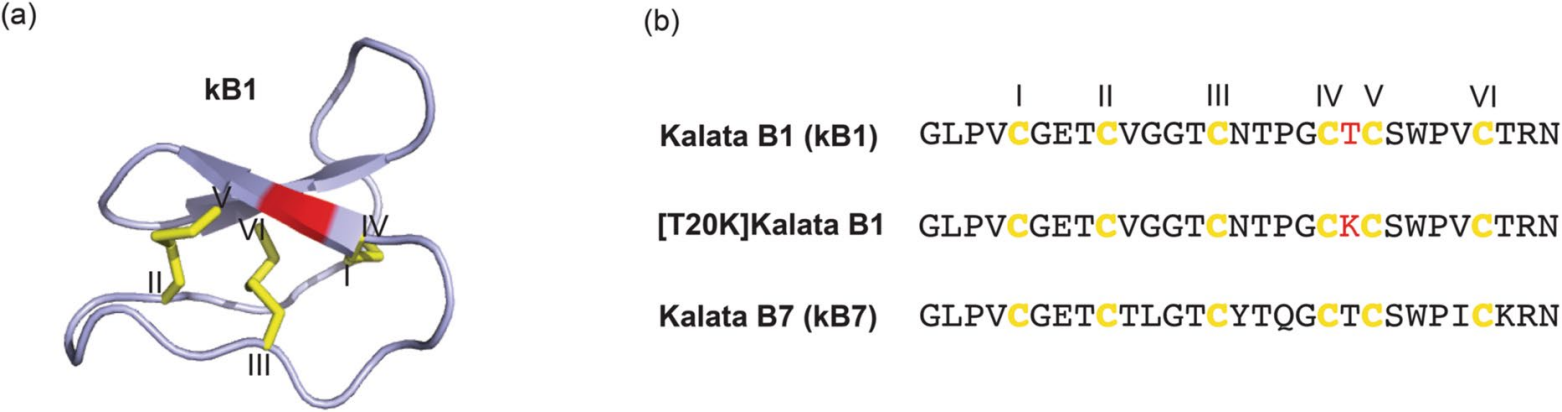
Sequences of selected cyclotides and 3D structure of the prototypic cyclotide kalata B1 (kB1) **a** Structure of kalata B1 highlighting the cyclic peptide backbone and cystine knot arrangement of the three disulfide bonds (PDB: 1NB1). The site of the T/K mutation to produce [T20K]kB1 is labelled in red. **b** Sequences of kalata B1, [T20K]kalata B1 and kalata B7 shown using one-letter amino acid codes.

One disadvantage of peptide-based drugs is their instability in the gastrointestinal system, with an almost universal requirement for administration by injection, a route not preferred by patients. In this context cyclotide-based drugs hold great potential due to their remarkable structural stability, mainly due to the presence of a cyclized backbone that incorporates a stabilizing cystine knot (Fig. 1a) (Craik et al. 1999). Indeed, the cyclotides kalata B1 and B7 (Fig. 1b) were found to be the only natural peptide scaffolds completely resistant to degradation in simulated gastric and intestinal digestion assays amongst a broad range of peptides tested (Kremsmayr et al. 2022). To determine the feasibility of oral dosing [T20K]kB1, Thell et al. (2016) compared the activity of parenteral and oral dosed [T20K]kB1 in a MS mouse model of experimental autoimmune encephalomyelitis (EAE). They found that, like the parenteral route, oral dosing significantly improved the EAE clinical score in a dose-dependent manner (10 and 20 mg/kg doses tested), highlighting the potential of [T20K]kB1 for use as a therapeutic administered by the patient-preferred oral delivery route.

With [T20K]kB1 now entering early stage clinical trials (Grundemann et al. 2019), it is timely to consider options for the cost-effective production of this peptide. Although many cyclotides are amenable to synthetic production using solid-phase peptide synthesis techniques, the cost of production at scale and the associated environmental footprint remains concerning (Andersson et al. 2000; Isidro-Llobet et al. 2019). Given that cyclotides are gene-encoded plant peptides, a recombinant plant-based production system is appealing. The biosynthetic pathway of cyclotides has been elucidated and it is well established that cyclotides mature through a series of proteolytic events (Jennings et al. 2001; Saska et al. 2007; Gillon et al. 2008; Rehm et al. 2019). Although subtle differences in the biosynthesis pathways exist between different cyclotide-producing species, one commonality is the requirement of a specific class of cysteine proteases, asparaginyl endopeptidases (AEPs), for backbone cyclization (Jackson et al. 2018, 2020). In proof-of-concept studies, using *Nicotiana benthamiana* as a biofactory plant, we have shown that by co-expressing specific ligase-capable AEPs with suitable peptide precursor genes, a range of diverse cyclic peptides can be produced in a scalable fashion (Jackson et al. 2022, 2018, 2019; Poon et al. 2018). Despite this success, one noteworthy limitation was for the production of the MS therapeutic candidate [T20K]kB1, which proved to be inefficiently produced *in planta* (Poon et al. 2018).

Here, we address the limited expression previously reported by testing a series of engineered [T20K]kB1 peptide precursor genes with the co-expression of a diverse set of AEP ligase genes. We find that subtle differences in both the peptide precursor design and the choice of co-expressed AEP ligase can greatly influence the yield of cyclized [T20K]kB1. By combining an optimized precursor and AEP ligase, we demonstrate that transient gene expression in *N. benthamiana* can yield up to 1 mg peptide per gram dry mass tissue in crude peptide extracts. The purified plant produced [T20K]kB1 was demonstrated by NMR spectroscopy to be structurally equivalent to synthetically produced peptide, paving the way for large-scale production of the therapeutic candidate [T20K]kB1.

## Materials and methods

### Vector construction

DNA encoding Oak1_T20K (OQ297794), Oak1_T20K_UTR (OQ297795), Oak1_T20K_NHIIAA (OQ297792), Oak1_T20K_NHIIAA_UTR (OQ297793), CterM_T20K (OQ297796) and NaD1_T20K (OQ297797) were synthesized by Integrated DNA Technologies (IDT) and delivered as gene blocks for cloning. Gene blocks were PCR amplified using primers designed with flanking *att*B sites for recombination into an intermediate vector PDS221 (Du et al. 2020), using Gateway BP Clonase (Invitrogen), and into pEAQ-DEST1 (Sainsbury et al. 2009), using LR Clonase II (Invitrogen). AEP expression vectors for OaAEP1b, OaAEP3, HeAEP3 and CtAEP1 have been previously described (Jackson et al. 2018). All vector sequences were validated by Sanger sequencing before transfer to *A. tumefaciens* strain LBA4404 for leaf infiltration experiments.

### Plant cultivation and infiltration

Wild type and ΔAEP *N. benthamiana* plants (Jackson et al. 2022) were grown hydroponically in a controlled environment growth room as part of the Clive and Vera Ramaciotti Facility for Producing Pharmaceuticals in Plants, situated at the University of Queensland, Australia. The temperature was set at 28 ^ο^C with 16 h light provided (170 μmol m^−2^ s^−1^ of LED illumination). At approximately 5-6 weeks of age, before flowering, plants were infiltrated with Agrobacteria using either needleless syringes (for small-scale assessment) or a vacuum chamber (for whole plants). For co-expression experiments, equal concentrations of Agrobacteria cultures (OD_600_ ~ 0.5) were mixed 1:1 in infiltration buffer (10 mM MES pH 5.6, 10 mM MgCl_2,_ 100 μM acetosyringone) and allowed to rest for up to 2 h before infiltration. At 5 days post infiltration, plants or leaf sections infiltrated were harvested, immediately snap frozen, and lyophilized for storage until processing.

### Peptide extraction and relative peptide quantification

Freeze-dried plant tissue was first ground to a powder using a GenoGrinder (SPEX SamplePrep) before total peptides were extracted using a 50% (v/v) acetonitrile, 1% (v/v) formic acid solution at a ratio of 50 μl solvent per mg of dry mass. After overnight incubation with gentle agitation, insoluble material was pelleted by centrifugation, and soluble peptide containing supernatant was retained. For relative peptide quantification, 10 μl of the supernatant (representing 0.5 mg dry mass) was mixed with 10 μl of an unrelated control peptide (GCCSDPRCNYDHPEICGGAAGN) and 80 μl of an 80% (v/v) acetonitrile, 1% (v/v) formic acid solution. The spiked peptide extracts were mixed 1:1 with α-cyano-4-hydroxycinnamic acid [5 mg ml^−1^ in 50% (v/v) acetonitrile / 0.1% trifluoroacetic acid (TFA) / 5 mM (NH_4_ H_2_PO_4_)] solution before being spotted onto a MALDI plate. MALDI-TOF-MS spectra data were acquired using a 5800 MALDI-TOF-MS (AB SCIEX) operating in reflector positive mode. For relative yield determination, the isotope cluster corresponding to the transgene-derived peptide was normalised to that obtained for the internally spiked peptide control.

### Purification of [T20K]kB1 and absolute peptide quantification

Whole plant tissue from six different plant batches were harvested at 5 days post vacuum-infiltration, then lyophilised overnight. Dry tissue was ground using a GenoGrinder (SPEX SamplePrep) with crude peptides extracted with a 50% (v/v) acetonitrile, 1% (v/v) formic acid solution at a ratio of 50 μl solvent per mg of dry mass. After gentle stirring overnight, the insoluble material was removed by centrifugation, and the soluble peptide was retained. To enable purification, supernatants containing crude peptides were lyophilized overnight and resuspended in a 10% (v/v) acetonitrile, 1% (v/v) formic acid solution for loading onto a Phenomenex Strata C18-E SPE cartridge with a 10 g resin capacity. A small aliquot of the eluted 10 – 80% (v/v) acetonitrile, 1% (v/v) acetonitrile fraction was retained for absolute quantification, while the remainder was lyophilised overnight and resuspended in 10% (v/v) acetonitrile, 0.1% (v/v) TFA in preparation for HPLC on a semi-preparative Phenomenex Jupiter C18 RP-HPLC column (250 × 10 mm, 5 μM particle size) connected to a Shimadzu LC-20AT pump system (Shimadzu Prominence). The purity of the *N. benthamiana* derived [T20K]kB1 was checked by MALDI-TOF–MS and with analytical HPLC using a C18 column (Phenomenex, Jupiter 5 μM, 150 × 2.0 mm).

Absolute [T20K]kB1 quantification of extracts and purified samples were performed using targeted multiple reaction monitoring (MRM) analyses conducted on a Sciex QTRAP® 6500^+^ mass spectrometer interfaced with an Exion UPLC system. Quantification methods were performed with a Phenomenex Kinetic C18 column (100 × 2.1 mm, 1.7 μm) at constant temperature of 60 °C using a linear acetonitrile gradient with a flow rate of 0.4 mL min^−1^. The source setting of electrospray voltage was set at 5500 V, curtain gas at 30, and temperature at 600 °C. MRM scans were performed with low resolution in Q1 targeting 973.9^3+^ and unit resolution in Q3 targeting 564.3^3+^ with delustering potential and collision energy set at 145 eV and 80 V, respectively. Synthetic [T20K]kB1 peptide was used to calculate a standard curve, of which unknown samples were plotted against.

### NMR spectroscopy

Purified [T20K]kB1 was dissolved in 550 µl of H2O/D2O (9:1) and the pH adjusted to 4.0. 1D proton and 2D TOCSY (80 ms mixing time) and NOESY (200 ms mixing time) spectra were acquired at 298 K on a Bruker Avance III 600 spectrometer equipped with a cryogenically cooled probe. Excitation sculpting methods were used to suppress solvent peaks and spectra were referenced internally to 4,4-dimethyl-4-silapentane-1-sulfonic acid at 0.0 ppm. All spectra were processed using TOPSPIN 3.6.1 (Bruker) and assignments made using using CCPNMR Analysis (version 2.4.4).

### Statistical analysis

ANOVA was performed using GraphPad Prism v9.5.0 software. Data sets were analysed using the nonparametric Kruskal–Wallis test with Dunn’s multiple comparisons. Significantly (p < 0.05) different treatment means are labelled with unique Greek letters in figures.

## Results

### Asparaginyl endopeptidase (AEP) ligases differ in their efficiency for the cyclization of [T20K]kB1

**Fig. 2:**
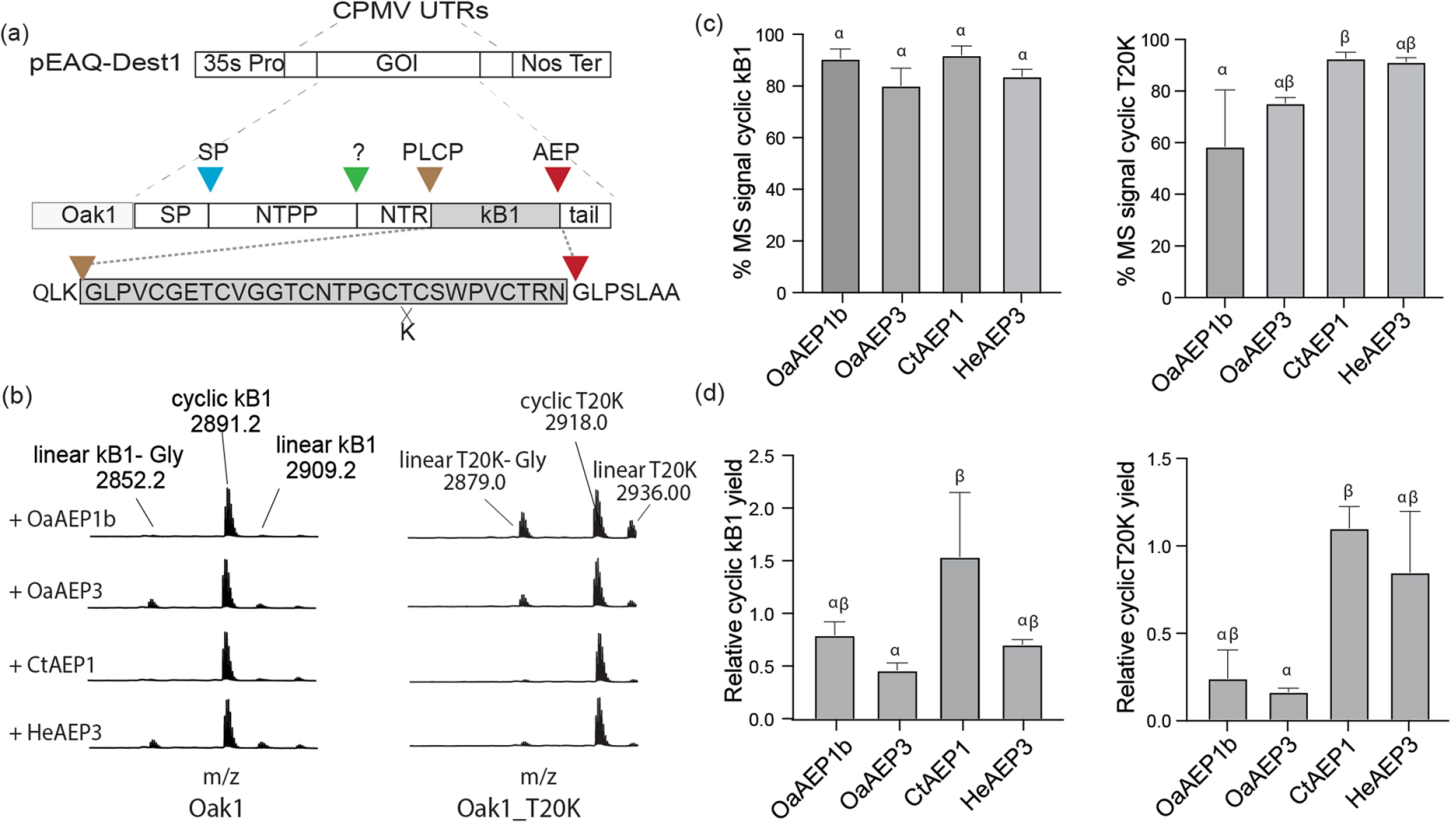
T20K peptide cyclization by co-expression of peptide precursors with ligase-type asparaginyl endopeptidases (AEPs). **a** AEP genes *OaAEP1b*, *OaAEP3*, *CtAEP1* and *HeAEP3* and *Oak1* precursor genes encoding kB1 or [T20K]kB1 were inserted into the plant expression vector pEAQ-DEST1. The Oak1 precursor is known to undergo sequential processing events to liberate the final cyclic peptide, including a signal peptide cleavage via a signal peptidase (SP) (blue triangle), N-terminal pro-peptide (NTPP) cleavage by an unknown protease (green triangle), N-terminal repeat (NTR) cleavage by a papain-like cysteine protease (brown triangle), and a final backbone cyclization by a ligase-type AEP (red triangle). **b** MALDI-MS-TOF assessment of peptides produced by co-expression of precursors Oak1 and Oak1_T20K with AEP ligases. For all four AEPs, predominant MS signals are evident for cyclic kB1 and [T20K]kB1 with varying degrees of N-terminal trimmed and full-length linear peptide. **c** Percent of MS signals representing correctly cyclized peptide (*n* = 4 s.d). Treatments carrying unique Greek letters are significantly different as determined by Kruskal–Wallis ANOVA. **d** Relative cyclic peptide yields as determined by normalizing cyclic peptide signal to that of an internally spiked peptide control (*n* = 4 s.d). Treatments carrying unique Greek letters are significantly different (*p* < 0.05) as determined by Kruskal–Wallis ANOVA. (Color figure online)

Our first aim was to identify an AEP ligase most favorable for [T20K]kB1 cyclization *in planta*. For this, we compared the expression of *Oak1_T20K*, encoding [T20K]kB1, and as a control *Oak1*, encoding wild type kB1, with the co-expression of four known AEP ligases, two from *O. affinis* (OaAEP1b and OaAEP3) (Harris et al. 2015, 2019), and one each from *Clitoria ternatea* (CtAEP1; butelase-1) (Nguyen et al. 2014) and *Hybanthus enneaspermus* (HeAEP3) (Jackson et al. 2018). For transient expression, precursor peptide and AEP genes were placed within the pEAQ-DEST1 vector (Sainsbury et al. 2009) (Fig. 2a), and infiltrated into *N. benthamiana* leaf cells using Agrobacterium. Five days post leaf infiltration, leaf tissue was sampled for peptide extraction and MALDI-MS analysis, where the resultant proportion of MS signals for AEP generated cyclic and linear peptides were calculated (Fig. 2b and c). We found that the Oak1 precursor was efficiently processed to cyclic kB1 by co-expression of all four chosen *AEP* ligase genes, with OaAEP1b and CtAEP1 the best performing at 91.2% ± 3.8% s.d and 92.7% ± 3.4% s.d of the total kB1 MS signal representing the mass for cyclic kB1 (Fig. 2b and c). For OaAEP3 and HeAEP3, the efficiency of cyclisation was marginally lower, with cyclic to linear peptide ratios of 80.8% ± 6.8% s.d and 84.4% ± 2.7% s.d respectively. In the case of Oak_T20K, cyclization efficiency was highest for CtAEP1 and HeAEP3 (93.2% ± 2.3% s.d and 91.7% ± 1.6% s.d respectively), and lower for the AEPs from *O. affinis*, with OaAEP1b co-expression resulting in 58.9% ± 21.9% s.d cyclic MS signal and OaAEP3 giving 75.7% ± 2.2% s.d. This low efficiency of OaAEP1b on the Oak_T20K substrate is in agreement with that previously reported by Poon et al. (2018).

Although calculating cyclic to linear peptide ratios provides a good indication of AEP functional preference (cyclization versus hydrolysis), it does not provide an assessment of total *in planta* enzyme performance, which is governed not only by AEP processing preference but also transcript stability, translational efficiency, enzyme maturation and turnover. To provide this measure, we quantified relative peptide levels by normalized resultant cyclic kB1 and [T20K]kB1 MS signals to that of a synthetic peptide standard that was spiked into peptide extracts on a per dry mass extract basis (Fig. 2d). This analysis indicated that CtAEP1 was the best performing AEP ligase for both the Oak1 and Oak1[T20K] substrates, with a ~ 2.0 and ~ 4.5 fold improvement in respective relative yields over OaAEP1b co-expression.

### The Oak1 precursor arrangement and associated 3’ UTR sequence enhance cyclotide yield

**Fig. 3:**
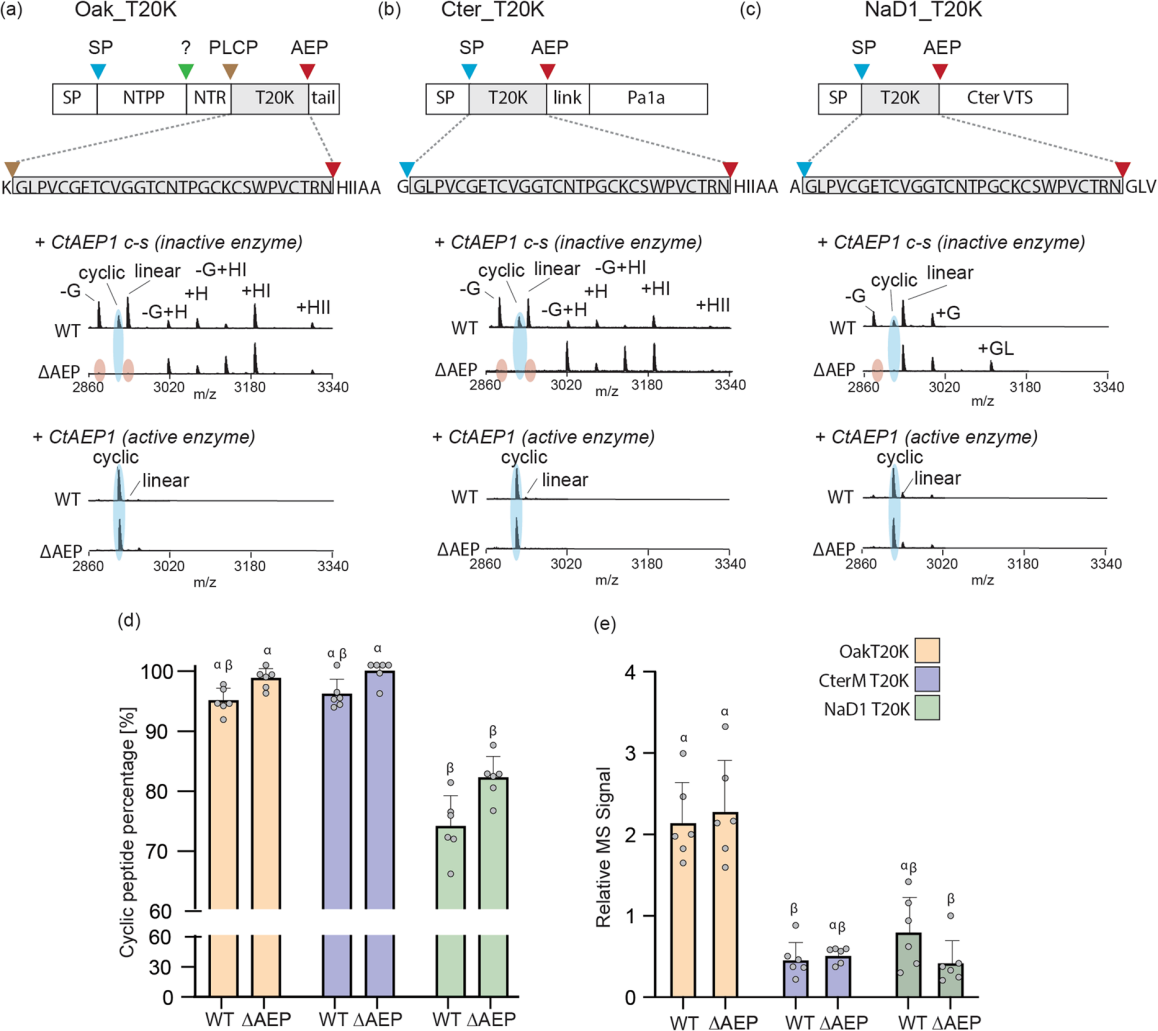
*In planta* assessment of peptide precursors for the production of [T20K]kB1. Precursors were engineered to encode for the T20K cyclotide domain and included **a** Oak_T20K, **b** Cter_T20K and **c** NaD1_T20K. While Oak_T20K is known to require several N-terminal processing steps (see Fig. 2), Cter_T20K and NaD1_T20K require only a signal peptidase (SP) event to liberate the N-terminus of [T20K]kB1, required for the AEP mediated backbone cyclisation event. Additional precursor domains include the Pa1a domain (precursor domain 1a) and the Cter VTS (C-terminal vacuole targeting signal). MALDI-MS-TOF analysis of crude peptide extracts revealed cyclic (blue highlight), linear and linear truncated or extended peptides as indicated. An absence of endogenous AEP-derived MS signals (highlighted in red) is notable when precursors are expressed with the catalytic inactive CtAEP1 c-s enzyme in the ΔAEP *N. benthamiana* line, when compared to wild type (WT) plants. **d** Percent of MS signals representing correctly cyclized peptide (*n* = 6 s.d). Treatments carrying unique Greek letters are significantly different (*p* < 0.05) as determined by Kruskal–Wallis ANOVA. (e) Relative cyclic [T20K]kB1 peptide yields as determined by normalizing the cyclic peptide signal to that of an internally spiked peptide control (*n* = 6 s.d). Treatments carrying unique Greek letters are significantly different (*p* < 0.05) as determined by Kruskal–Wallis ANOVA

Although cyclotides are highly conserved across the five plant families known to produce them, their respective precursor propeptides carry unique differences in the arrangement of their pro-peptide domains and subcellular targeting sequences, which ultimately may influence the yield of a heterologous processed peptide. To determine if we could improve upon the Oak1_T20K precursor (Fig. 3a), we prepared two additional T20K peptide precursor genes, one based on the precursor of the Fabaceae cyclotide CterM (Fig. 3b), and the other the defensin peptide NaD1 from *N. tabacum* (Fig. 3c). Like Oak1, each of these precursors is predicted to enter the endomembrane system of plant cells by virtue of a signal peptide, and also predicted to transit to the vacuole compartment for peptide maturation (Lay et al. 2014). In the case of Oak1_T20K, we made one additional change where the peptide’s C-terminal tailing sequence GLPSLAA was replaced with HIIAA, as is present in the CterM precursor. For this analysis, we co-expressed each precursor with a *N. benthamiana* codon-optimized gene sequence variant of CtAEP1 (CtAEP1_nbOPT), and as a negative control, a catalytically inactive mutant CtAEP1 (C207-S). Both wild-type *N. benthamiana* and our recently developed ΔAEP (a quad AEP knockout mutant line (Jackson et al. 2022)) were infiltrated, with the latter included to determine if yields were improved in this reduced endogenous AEP background.

Irrespective of precursor, co-expression of the catalytic inactive CtAEP1 (207C-S) mutant resulted in predominantly linear peptides produced (full length, N-terminal truncated and C-terminal extended forms), as determined by MALDI-MS-TOF (Fig. 3a-c). In wild-type *N. benthamiana* plants, the majority of these linear peptides are evidently processed products from endogenous AEPs that lack the functional ability to efficiently cyclize the [T20K]kB1 backbone, supported by the complete absence of such processed peptides in the ΔAEP background. Upon co-expression of the catalytically active CtAEP1_nbOPT, all precursors were predominantly processed to cyclic [T20K]kB1, with similar cyclic to linear peptide ratios observed for Oak1_T20K (94.2% ± 2.1% s.d.) and CterM_T20K (95.2% ± 2.5% s.d.), while a lower percentage was recorded for NaD1_T20K (73.5% ± 5.1% s.d.) (Fig. 3d). Expression within the ΔAEP line produced almost undetectable linear peptide for Oak1_T20K and CterM_T20K with cyclic peptide percentage at 97.8% ± 1.7% s.d. and 99.0% ± 1.9% s.d. respectively. NaD1_T20K cyclic peptide percentage was also increased in the ΔAEP line, to 81.5% ± 3.5% s.d. Relative cyclic [T20K]kB1 levels were also determined with the highest yields obtained for the Oak1_T20K construct, which produced ~ 2.6 fold higher levels than the next best NaD1_T20K precursor (Fig. 3e). Despite improved cyclic to linear percentages observed upon expression in the ΔAEP line (Fig. 3d), only marginally improved cyclic peptide levels were calculated (Fig. 3e).

**Fig. 4:**
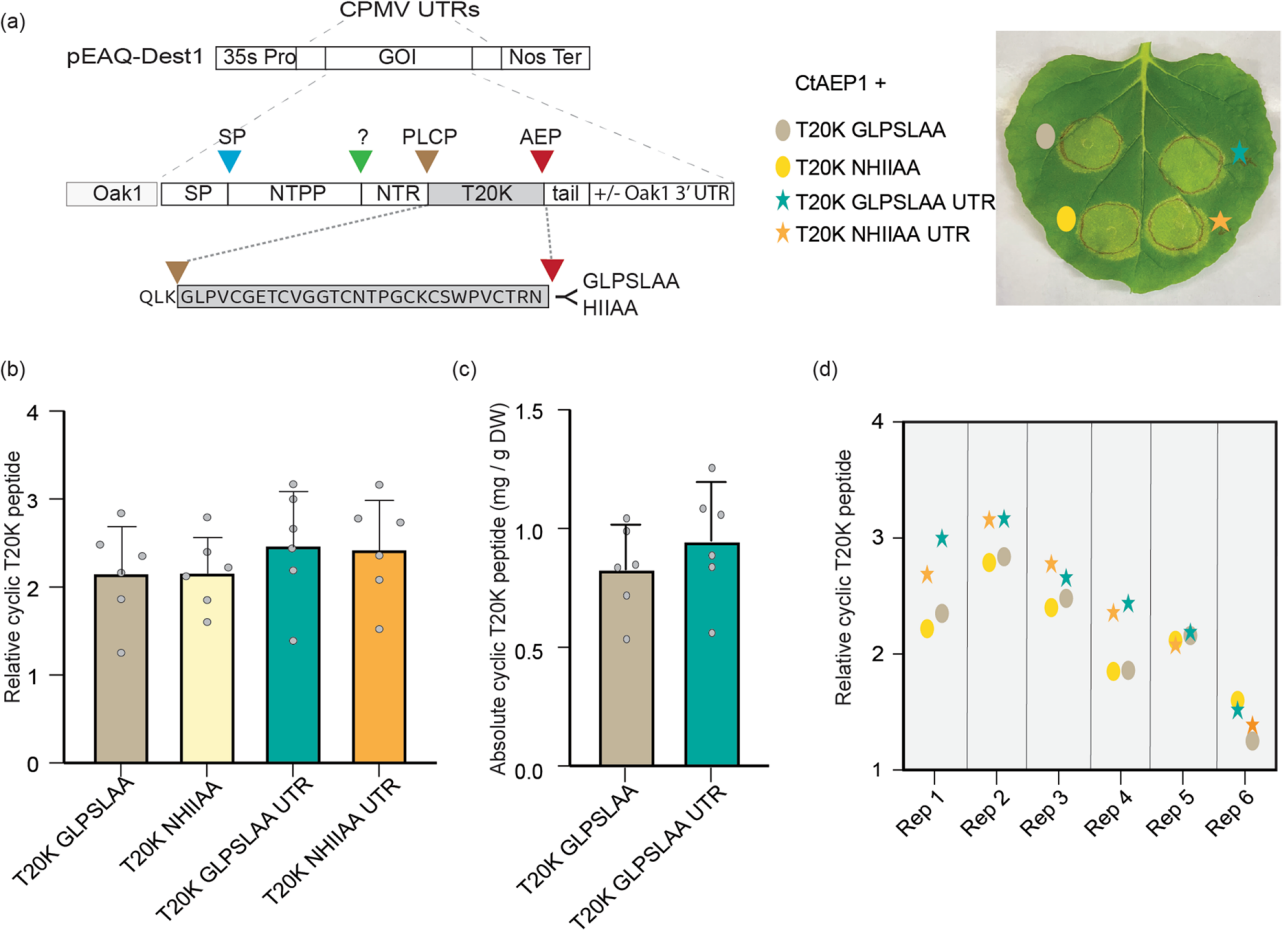
Relative yield effects by altering AEP recognition sequences and providing an additional 3’ UTR sequence. **a** Oak_T20K precursors were designed to include either a GLPSLAA tail, as naturally present in Oak1, or a HIIAA tail, as naturally present within the interdomain linker of CterM. Both variants were tested with and without the inclusion of the 3’ UTR region of Oak1. All four expression constructs were co-expressed with CtAEP1. **b** Relative cyclic [T20K]kB1 peptide yields as determined by normalizing the cyclic peptide MS signal to that of an internally spiked peptide control (*n* = 6 s.d). **c** Absolute quantification was performed for T20K GLPSLAA with and without the UTR sequence (*n* = 6 s.d). **d** Replicate plant data showing relative yield on individual leaves

Having established that the Oak1 precursor framework combined with CtAEP1_nbOPT is the most favorable combination for [T20K]kB1 production *in planta*, we next tested other variables that we considered could influence yields. In the first instance, we added in 268 nt 3’UTR of the native Oak1 transcript. In effect, this arrangement provides for a tandem 3’ UTR as the pEAQ vector also carries the 3’ UTR of the CPMV followed by the Nos terminator sequence (Fig. 4a) (Sainsbury et al. 2009). Additionally, we performed a side-by-side comparison of Oak1_T20K constructs varying in their C-terminal tailing residues (GLPSLAA vs HIIAA). We used small scale infiltrations to express each of these four precursors with CtAEP1_nbOPT, and we calculated relative [T20K]kB1 peptide produced (Fig. 4b). Although great care was used to ensure leaves infiltrated were of the same developmental age, we found substantial variation in relative expression yield across the six replicate infiltrations (Fig. 4b). When each leaf was analyzed independently, the highest yielding infiltration spots were largely from constructs carrying the 3’UTR sequence of *Oak1*, irrespective of the precursor harboring GLPSLAA or NHIIAA tails. This finding suggests that the 3’UTR of *Oak1* enhances the final yield of [T20K]kB1 (Fig. 4d).

To corroborate our relative quantifications, we determined absolute [T20K]kB1 quantifications of peptide extracts from Oak1[T20K] GLPSLAA with and without the Oak 3’ UTR (Fig. 4c). Like the relative quantifications, absolute peptide levels were routinely higher when the 3’ UTR was included in the expression construct. In these crude peptide extracts, yields for Oak1[T20K] GLPSLAA with and without the inclusion of the Oak1 3’ UTR sequences were calculated as 0.95 ± 0.24 s.d and 0.83 ± 0.19 s.d mg/g dry mass tissue. For comparison purposes (and based on historic fresh mass to dry mass measurements), this yield would equate to roughly 0.21 and 0.18 mg/g fresh mass respectively.

### Structural equivalence of plant-derived [T20K]kB1

**Fig. 5:**
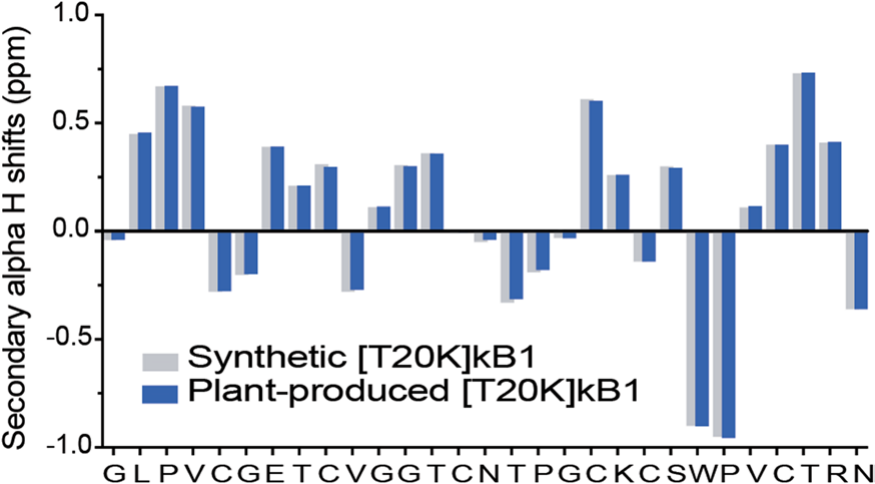
Structural equivalence of plant-derived and synthetic T20K. Secondary Hα chemical shift comparison of [T20K]kB1 produced by solid phase peptide chemistry (synthetic) with the plant produced peptide purified from *N. benthamiana* leaf

We next aimed to produce [T20K]kB1 at scale to test purification and provide pure peptide for NMR structural analysis. For this, we co-expressed our best performing precursor Oak_T20K GLPSLAA UTR and AEP ligase pEAQ CtAEP1_nbOPT in wild type *N.benthamiana*. Across six batches of plants infiltrated, absolute quantification of [T20K]kB1 in crude peptide extracts averaged 0.30 ± 0.07 mg/g dry mass. In order to purify [T20K]kB1 for NMR analysis, crude peptides from three different batches were combined and purified using gravity fed C18 solid phase extraction (SPE), followed by reverse phase HPLC. Resultant yield losses were recorded through purification, revealing a 59% loss (~ 4.5 mg to 1.86 mg) of peptide after C18 SPE, and an additional 65% loss (1.86 mg to 0.65 mg) during HPLC. The resulting plant produced [T20K]kB1 was sufficiently pure (Fig S1a) for NMR structural analysis (Fig S1b). Calculation of secondary Hα shifts (i.e. the difference of measured chemical shifts from random coil values (Wishart et al. 1995) and comparison with synthetic-derived peptide indicate an identical backbone structure and secondary structural elements (Fig. 5), confirming cyclisation and correct folding of the plant derived peptide.

## Discussion

Given their exceptional stability, cyclotides have attracted significant interest as scaffolds for drug development (Camarero and Campbell 2019; Craik et al. 2012). With a pipeline of cyclotide-based therapeutic candidates in development (de Veer et al. 2019), it is timely to consider a means of recombinant production that is both environmentally friendly and economically favored over traditional synthetic peptide production methods (Isidro-Llobet et al. 2019; Andersson et al. 2000). In this study we investigated the feasibility of using the biofactory plant *N. benthamiana* for the rapid and scalable production of the MS therapeutic candidate [T20K]kB1, that has advanced to early stage clinical trials as a treatment to reduce the symptoms of multiple sclerosis (Grundemann et al. 2019).

Efforts to unravel the cyclotide biosynthesis pathway in plants have led to the identification of a class of AEPs that function efficiently in the backbone cyclisation of diverse cyclotides (Harris et al. 2015; Jackson et al. 2018, 2020; Du et al. 2020; Nguyen et al. 2014). These ligase-type AEPs, alternatively termed peptide asparaginyl ligases (PALs) (Hemu et al. 2019), are rare in nature and have predominantly been characterized from cyclotide-producing plant species (Hemu et al. 2022). Bioinformatic and structural activity studies have identified a number of subtle structural features of AEP ligases that steer AEP activity towards peptide ligation over hydrolysis (Hemu et al. 2022, 2020, 2019; Jackson et al. 2018; Yang et al. 2017). Here we demonstrate that subtle changes in the substrate can additionally influence AEP ligase efficiency, with the T20K substitution negatively affecting the processing ability of the OaAEP1b ligase from *O. affinis.* This amino acid substitution is some distance from the required AEP recognition tripeptide sequence, NGL; thus, we propose that this drop in efficiency is caused by enzyme substrate repulsion due to the addition of this charged residue. In contrast, CtAEP1 activity *in planta* was equally efficient on both wild type kB1 and T20K[kB1] substrates, suggesting that this enzyme is more tolerant of the residue exchange, while HeAEP3 slightly favored the T20K[kB1] substrate over the wild type kB1. With these unpredictable enzyme efficiencies, it is pertinent to always test a panel of AEP ligases for identification of the best *in planta* performing enzyme. Importantly, such an assay is relatively quick using transient expression in *N. benthamiana*.

Cyclotides, like most plant-derived peptides, are encoded within larger precursor proteins that require proteolytic processing (Jennings et al. 2001; Gillon et al. 2008; Conlan et al. 2011, 2012). One commonality across all cyclotide precursors, regardless of the plant family from which they originate, is the presence of a plant family-specific AEP processing site at the peptide C-terminus. By contrast, the mechanism for release of the cyclotide N-terminus differs. For cyclotides from the Fabaceae, only a signal peptide cleavage is required to liberate the N-terminus, whilst for those from the Rubiaceae, Violaceae and Solanaceae, a dedicated N-terminal processing enzyme is required, with a papain-like cysteine protease implicated for the Oak1 precursor (Rehm et al. 2019). Here, we wished to determine if we could improve the *in planta* yield of cyclic [T20K]kB1 by engineering processing of the peptide from the precursor proteins of the cyclotide CterM and the defensin NaD1 from ornamental tobacco (Lay et al. 2003). When each of these three precursors was co-expressed with CtAEP1, correctly cyclized [T20K]kB1 predominated the MALDI-MS profile, indicating that CtAEP1 and the tested precursor proteins correctly co-localize for AEP-mediated backbone cyclisation of [T20K]kB1 to occur, most likely within the plant cell vacuole where AEPs are thought to self-activate (Hara-Nishimura et al. 1998). Despite similar cyclic to linear peptide ratios, the yield of CterM_T20K and NaD1_T20K were both lower than the Oak1_T20K precursor arrangement. Although the reasons for this are not immediately evident, efficiencies in N-terminal cleavage (signal peptide mediated versus PLCP mediated) and in vacuole targeting (C-terminal versus N-terminal signals) may be influential.

The pEAQ vector used in this study for transient expression of *N. benthamiana* provides for high-level transgene expression due to a unique arrangement of a cowpea mosaic virus (CMV) RNA-2 5’ UTR and a chimeric 3’ UTR, that includes 183 nucleotides of the CMV RNA-2 3’ UTR linked to the Nos terminator sequence (Sainsbury et al. 2009). While the modified 5’UTR has been demonstrated to enhance protein translation (Sainsbury and Lomonossoff 2008), the chimeric 3’ UTR enhances mRNA accumulation (Meshcheriakova et al. 2014). As the *Oak1* gene is one of the most highly expressed genes in *O. affinis* leaf (Qin et al. 2010), we were interested in testing whether the inclusion of the 3’UTR could further enhance [T20K]kB1 production levels. We observed that, despite using similarly aged leaves, yields varied significantly across experiments, limiting the interpretation of yield data (Fig. 4b, c). However, when considering each replicate leaf (spot infiltrated with each of the four expression vectors), it is notable that the expression vectors carrying the additional 3’ UTR of *Oak1* provided a greater yield in 4 of the 6 plants tested (Fig. 4d). Further work is required to confirm this positive effect and test whether other recombinant products would benefit from this chimeric 3’ UTR.

Beyond optimizing plant-based expression levels, downstream processing that is simple, cost-effective, and efficient is paramount. Whereas most attempts at using plants for recombinant expression of peptides have relied on the expression of fusion proteins, with a latter requirement for in vitro processing (Ghidey et al. 2020), here we use plant enzymes (endogenous and transgene derived) for a one-step in vivo maturation of cyclic peptide. In this way, mature [T20K]kB1 can be directly purified based on its hydrophobicity using reversed-phase HPLC. By calculating [T20K]kB1 yields in crude peptide extracts and through purification steps, we found that although purification using C18 RP-HPLC was robust for achieving highly pure peptide (Fig S1a), it resulted in considerable peptide loss (~ 85%). A study by Kristensen et al. (2015) revealed that this phenomenon is rather common for cationic peptides and is due to the adsorption of peptides to solid plastic and glass surfaces. In future studies, we will devise strategies to reduce this phenomenon, including using of pre-fabricated low protein binding tubes, maximizing peptide concentration at all times, and possibly even using plastics and glass with pre-saturated surfaces.

The development of plants as environmentally friendly and scalable production vehicles for recombinant proteins is building momentum (Huebbers and Buyel 2021). In the current study, we tested a range of variables to optimize the production of the MS therapeutic candidate [T20K]kB1 in *N. benthamiana*. Influential was the choice of co-expressed AEP ligase, the precursor protein arrangement, and the inclusion of UTR sequences in the expression vector. Laboratory scale whole plant infiltrations yielded on average 0.3 mg/g dry mass of correctly cyclized [T20K]kB1, with over threefold this amount quantified from isolated infiltration spots of younger leaves. With further improvements in downstream processing and production at scale, we anticipate that plant-based production of therapeutic cyclic peptides is now within reach.

## Supplementary Information

Below is the link to the electronic supplementary material.

Supplementary file1 (DOCX 84 KB)

## References

[CR1] Andersson L, Blomberg L, Flegel M, Lepsa L, Nilsson B, Verlander M (2000). Large-scale synthesis of peptides. Biopolymers.

[CR2] Camarero JA, Campbell MJ (2019). The potential of the cyclotide scaffold for drug development. Biomedicines.

[CR3] Conlan BF, Gillon AD, Barbeta BL, Anderson MA (2011). Subcellular targeting and biosynthesis of cyclotides in plant cells. Am J Bot.

[CR4] Conlan BF, Colgrave ML, Gillon AD, Guarino R, Craik DJ, Anderso MA (2012). Insights into processing and cyclization events associated with biosynthesis of the cyclic peptide Kalata B1. J Biol Chem.

[CR5] Craik DJ, Daly NL, Bond T, Waine C (1999). Plant cyclotides: a unique family of cyclic and knotted proteins that defines the cyclic cystine knot structural motif. J Mol Biol.

[CR6] Craik DJ, Swedberg JE, Mylne JS, Cemazar M (2012). Cyclotides as a basis for drug design. Expert Opin Drug Discov.

[CR7] de Veer SJ, Kan MW, Craik DJ (2019). Cyclotides: from structure to function. Chem Rev.

[CR8] Du JQ, Yap K, Chan LY, Rehm FBH, Looi FY, Poth AG, Gilding EK, Kaas Q, Durek T, Craik DJ (2020). A bifunctional asparaginyl endopeptidase efficiently catalyzes both cleavage and cyclization of cyclic trypsin inhibitors. Nature Commun.

[CR9] Ghidey M, Islam SMA, Pruett G, Kearney CM (2020). Making plants into cost-effective bioreactors for highly active antimicrobial peptides. New Biotechnol.

[CR10] Gillon AD, Saska I, Jennings CV, Guarino RF, Craik DJ, Anderson MA (2008). Biosynthesis of circular proteins in plants. Plant J.

[CR11] Grundemann C, Koehbach J, Huber R, Gruber CW (2012). Do plant cyclotides have potential as immunosuppressant peptides?. J Nat Prod.

[CR12] Grundemann C, Thell K, Lengen K, Garcia-Kaufer M, Huang YH, Huber R, Craik DJ, Schabbauer G, Gruber CW (2013). Cyclotides Suppress Human T-Lymphocyte Proliferation by an Interleukin 2-Dependent Mechanism. Plos One.

[CR13] Grundemann C, Stenberg KG, Gruber CW (2019). T20K: an Immunomodulatory cyclotide on its way to the clinic. Int J Pept Res Ther.

[CR14] Hara-Nishimura I, Kinoshita T, Hiraiwa N, Nishimura M (1998). Vacuolar processing enzymes in protein-storage vacuoles and lytic vacuoles. J Plant Physiol.

[CR15] Harris KS, Durek T, Kaas Q, Poth AG, Gilding EK, Conlan BF, Saska I, Daly NL, van der Weerden NL, Craik DJ, Anderson MA (2015). Efficient backbone cyclization of linear peptides by a recombinant asparaginyl endopeptidase. Nature Commun.

[CR16] Harris KS, Guarino RF, Dissanayake RS, Quimbar P, McCorkelle OC, Poon S, Kaas Q, Durek T, Gilding EK, Jackson MA, Craik DJ, van der Weerden NL, Anders RF, Anderson MA (2019). A suite of kinetically superior AEP ligases can cyclise an intrinsically disordered protein. Sci Rep.

[CR17] Hellinger R, Muratspahić E, Devi S, Koehbach J, Vasileva M, Harvey PJ, Craik DJ, Gründemann C, Gruber CW (2021). Importance of the cyclic cystine knot structural motif for immunosuppressive effects of cyclotides. ACS Chem Biol.

[CR18] Hemu XY, El Sahili A, Hu SD, Wong KH, Chen Y, Wong YH, Zhang XH, Serra A, Goh BC, Darwis DA, Chen MW, Sze SK, Liu CF, Lescar J, Tam JP (2019). Structural determinants for peptide-bond formation by asparaginyl ligases. Proc Natl Acad Sci USA.

[CR19] Hemu X, El Sahili A, Hu S, Zhang X, Serra A, Goh BC, Darwis DA, Chen MW, Sze SK, Liu C-f, Lescar J, Tam JP (2020). Turning an asparaginyl endopeptidase into a peptide ligase. ACS Catal.

[CR20] Hemu X, Chan N-Y, Liew HT, Hu S, Zhang X, Serra A, Lescar J, Liu C-F, Tam JP (2022) Substrate-binding glycine residues are major determinants for hydrolase and ligase activity of plant legumains. bioRxiv doi: 10.1101/2022.09.26.509423 10.1111/nph.18841 36843268

[CR21] Huebbers JW, Buyel JF (2021). On the verge of the market – Plant factories for the automated and standardized production of biopharmaceuticals. Biotechnol Adv.

[CR22] Isidro-Llobet A, Kenworthy MN, Mukherjee S, Kojach ME, Wegner K, Gallou F, Smith AG, Roschangar F (2019). Sustainability challenges in peptide synthesis and purification: from R&D to production. J Org Chem.

[CR23] Jackson MA, Gilding EK, Shafee T, Harris KS, Kaas Q, Poon S, Yap K, Jia H, Guarino R, Chan LY, Durek T, Anderson MA, Craik DJ (2018). Molecular basis for the production of cyclic peptides by plant asparaginyl endopeptidases. Nature Commun.

[CR24] Jackson MA, Yap K, Poth AG, Gilding EK, Swedberg JE, Poon S, Qu H, Durek T, Harris K, Anderson MA, Craik DJ (2019). Rapid and scalable plant-based production of a potent plasmin inhibitor peptide. Front Plant Sci.

[CR25] Jackson MA, Nguyen LTT, Gilding EK, Durek T, Craik DJ (2020). Make it or break it: Plant AEPs on stage in biotechnology. Biotechnol Adv.

[CR26] Jackson MA, Chan LY, Harding MD, Craik DJ, Gilding EK (2022). Rational domestication of a plant-based recombinant expression system expands its biosynthetic range. J Exp Bot.

[CR27] Jarmolowicz DP, Bruce AS, Glusman M, Lim SL, Lynch S, Thelen J, Catley D, Zieber N, Reed DD, Bruce JM (2017). On how patients with multiple sclerosis weigh side effect severity and treatment efficacy when making treatment decisions. Exp Clin Psychopharmacol.

[CR28] Jennings C, West J, Waine C, Craik D, Anderson M (2001). Biosynthesis and insecticidal properties of plant cyclotides: the cyclic knotted proteins from Oldenlandia affinis. Proc Natl Acad Sci USA.

[CR29] Kremsmayr T, Aljnabi A, Blanco-Canosa JB, Tran HNT, Emidio NB, Muttenthaler M (2022). On the utility of chemical strategies to improve peptide gut stability. J Med Chem.

[CR30] Kristensen K, Henriksen JR, Andresen TL (2015). Adsorption of cationic peptides to solid surfaces of glass and plastic. Plos One.

[CR31] Lay FT, Brugliera F, Anderson MA (2003). Isolation and properties of floral defensins from ornamental tobacco and petunia. Plant Physiol.

[CR32] Lay FT, Poon S, McKenna JA, Connelly AA, Barbeta BL, McGinness BS, Fox JL, Daly NL, Craik DJ, Heath RL, Anderson MA (2014). The C-terminal propeptide of a plant defensin confers cytoprotective and subcellular targeting functions. BMC Plant Biol.

[CR33] Leffler J, Trend S, Gorman S, Hart PH (2022). Sex-specific environmental impacts on initiation and progression of multiple sclerosis. Front Neurol.

[CR34] Macaron G, Ontaneda D (2019). Diagnosis and management of progressive multiple sclerosis. Biomedicines.

[CR35] Manouchehrinia A, Huang JS, Hillert J, Alfredsson L, Olsson T, Kockum I, Constantinescu CS (2022). Smoking attributable risk in multiple sclerosis. Front Immunol.

[CR36] McGinley MP, Goldschmidt CH, Rae-Grant AD (2021). Diagnosis and treatment of multiple sclerosis a review. Jama-J Am Med Assoc.

[CR37] Meshcheriakova YA, Saxena P, Lomonossoff GP (2014). Fine-tuning levels of heterologous gene expression in plants by orthogonal variation of the untranslated regions of a nonreplicating transient expression system. Plant Biotechnol J.

[CR38] Moiola L, Rommer PS, Zettl UK (2020). Prevention and management of adverse effects of disease modifying treatments in multiple sclerosis. Curr Opin Neurol.

[CR39] Morrow SA, Clift F, Devonshire V, Lapointe E, Schneider R, Stefanelli M, Vosoughi R (2022). Use of natalizumab in persons with multiple sclerosis: 2022 update. Multiple Sclerosis and Related Dis.

[CR40] Nguyen GKT, Wang SJ, Qiu YB, Hemu X, Lian YL, Tam JP (2014). Butelase 1 is an Asx-specific ligase enabling peptide macrocyclization and synthesis. Nat Chem Biol.

[CR41] Perez-Perez S, Dominguez-Mozo MI, Garcia-Martinez MA, Ballester-Gonzalez R, Nieto-Ganan I, Arroyo R, Alvarez-Lafuente R (2022). Epstein-barr virus load correlates with multiple sclerosis-associated retrovirus envelope expression. Biomedicines.

[CR42] Poon S, Harris KS, Jackson MA, McCorkelle OC, Gilding EK, Durek T, Van Der Weerden NL, Craik DJ, Anderson MA (2018). Co-expression of a cyclizing asparaginyl endopeptidase enables efficient production of cyclic peptides in planta. J Exp Bot.

[CR43] Pournajaf S, Dargahi L, Javan M, Pourgholami MH (2022). Molecular pharmacology and novel potential therapeutic applications of fingolimod. Front Pharmacol.

[CR44] Qin QP, McCallum EJ, Kaas Q, Suda J, Saska I, Craik DJ, Mylne JS (2010). Identification of candidates for cyclotide biosynthesis and cyclisation by expressed sequence tag analysis of Oldenlandia affinis. BMC Genom.

[CR45] Rehm FBH, Jackson MA, De Geyter E, Yap K, Gilding EK, Durek T, Craik DJ (2019). Papain-like cysteine proteases prepare plant cyclic peptide precursors for cyclization. Proc Natl Acad Sci USA.

[CR46] Sainsbury F, Lomonossoff GP (2008). Extremely high-level and rapid transient protein production in plants without the use of viral replication. Plant Physiol.

[CR47] Sainsbury F, Thuenemann EC, Lomonossoff GP (2009). pEAQ: versatile expression vectors for easy and quick transient expression of heterologous proteins in plants. Plant Biotechnol J.

[CR48] Saska I, Gillon AD, Hatsugai N, Dietzgen RG, Hara-Nishimura I, Anderson MA, Craik DJ (2007). An asparaginyl endopeptidase mediates in vivo protein backbone cyclization. J Biol Chem.

[CR49] Thell K, Hellinger R, Sahin E, Michenthaler P, Gold-Binder M, Haider T, Kuttke M, Liutkeviciute Z, Goransson U, Grundemann C, Schabbauer G, Gruber CW (2016). Oral activity of a nature-derived cyclic peptide for the treatment of multiple sclerosis. Proc Natl Acad Sci USA.

[CR50] Walther EU, Dietrich E, Hohlfeld R (1996). Therapy for multiple sclerosis with interferon-beta-1b. Advice to patients, including how to deal with side effects. Nervenarzt.

[CR51] Wishart DS, Bigam CG, Holm A, Hodges RS, Sykes BD (1995). 1H, 13C and 15N random coil NMR chemical shifts of the common amino acids I Investigations of nearest-neighbor effects. J Biomol NMR.

[CR52] Yang RL, Wong YH, Nguyen GKT, Tam JP, Lescar J, Wu B (2017). Engineering a catalytically efficient recombinant protein ligase. J Am Chem Soc.

