# Supplementary figures and images for: Plant-based production of an orally active cyclotide for the treatment of multiple sclerosis
Source: Transgenic Res. 2023 Mar 17;32(1-2):121–33. doi: 10.1007/s11248-023-00341-1 (PMC10102037; doi:10.1007/s11248-023-00341-1)

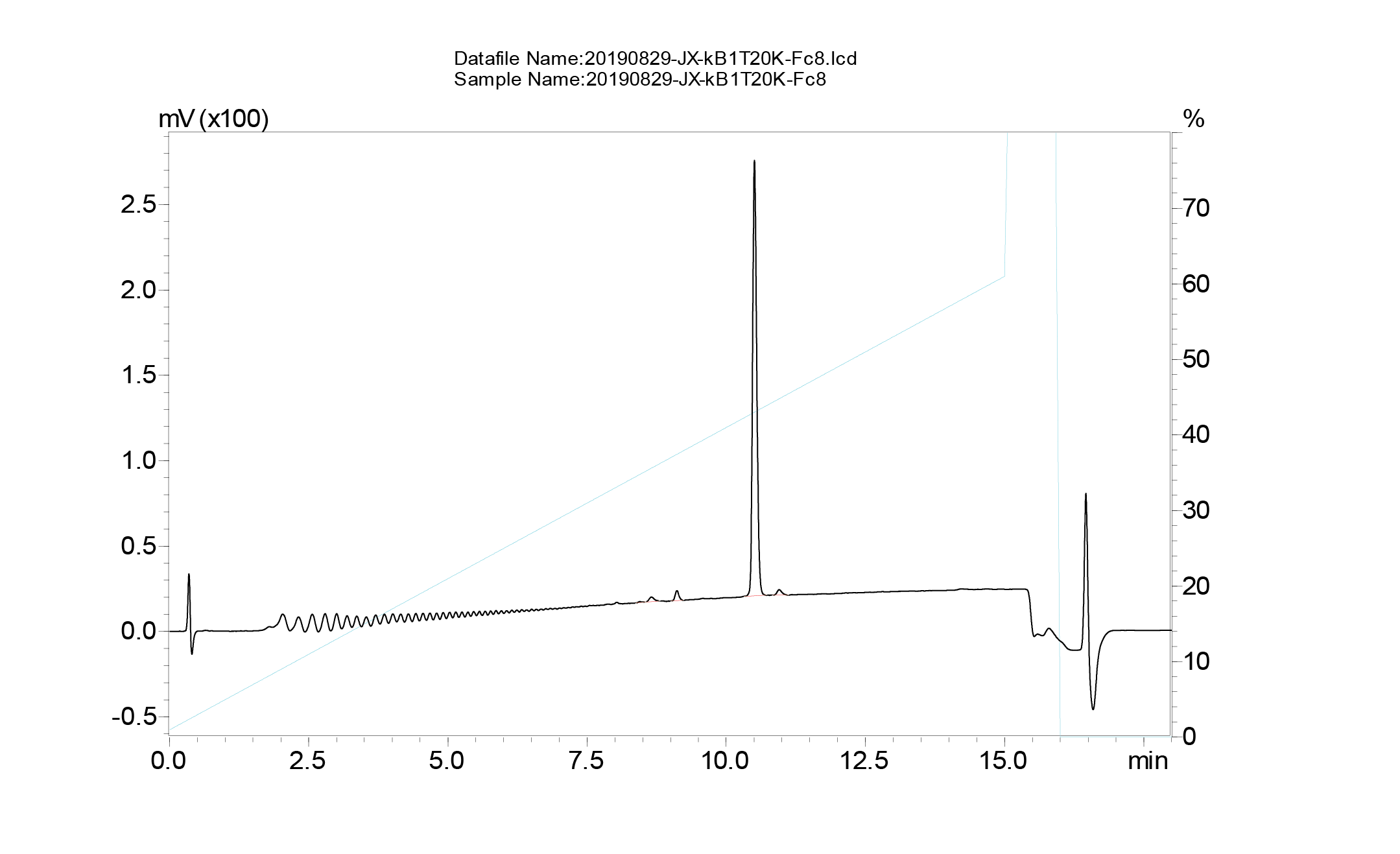


**Fig S1. UHPLC analysis of *N. benthamiana* derived [T20K]kB1 peptide.**

Supplement: Supplementary file 1 — Supplementary file1 (DOCX 84 KB) [file 11248_2023_341_MOESM1_ESM.docx]
